# Unusual Childhood Waking as a Possible Precursor of the 1995 Kobe Earthquake

**DOI:** 10.3390/ani3010228

**Published:** 2013-03-05

**Authors:** Motoji Ikeya, Neil E. Whitehead

**Affiliations:** 1 Quantum Geophysics Laboratory, Department of Earth and Space Sciences, University of Osaka, 1-1 Machikaneyama, Toyonaka, Osaka 560-0043, Japan; 2 Whitehead Associates, 54 Redvers Drive, Lower Hutt, 5010, New Zealand

**Keywords:** Earthquakes, Kobe, Hanshin, children, sleep, precursors, Rn-222

## Abstract

**Simple Summary:**

The paper investigates whether young children may waken before earthquakes through a cause other than foreshocks. It concludes there is statistical evidence for this, but the mechanism best supported is anxiety produced by Ultra Low Frequency (ULF) electromagnetic waves.

**Abstract:**

Nearly 1,100 young students living in Japan at a range of distances up to 500 km from the 1995 Kobe M7 earthquake were interviewed. A statistically significant abnormal rate of early wakening before the earthquake was found, having exponential decrease with distance and a half value approaching 100 km, but decreasing much slower than from a point source such as an epicentre; instead originating from an extended area of more than 100 km in diameter. Because an improbably high amount of variance is explained, this effect is unlikely to be simply psychological and must reflect another mechanism—perhaps Ultra-Low Frequency (ULF) electromagnetic waves creating anxiety—but probably not ^222^Rn excess. Other work reviewed suggests these conclusions may be valid for animals in general, not just children, but would be very difficult to apply for practical earthquake prediction.

## 1. Introduction

Observations of apparent precursory animal reaction to earthquakes have been so common throughout history that they have passed into folk proverbs. Examples of this include the premonitory call of the pheasant (ancient Persia) and the reactions of Japanese catfish. Explanatory mechanisms were not generally proposed in this pre-scientific era.

Tributsch [1] reported many current instances of apparent animal reactions to forthcoming earthquakes. He theorized that the cause was aerosols produced from the ground by precursory strains, and further, that the serotonin system in animals mediated this. The degree of detail supporting this theory was not matched until the work of Ikeya [2].

Many researchers have reported or theorized the presence of higher ^222^Rn levels before earthquakes (e.g., [3]). However there appear to be no papers showing that animals are capable of detecting the gas.

Kirschvink [4] proposed detection of geomagnetic changes mediated by known magnetite occurrence in tissues of animals as one possible earthquake precursor. However his main focus was whale strandings. Animals tested in other laboratories showed undetectable responses to strong, static magnetic fields, and the very large magnetic fields of MRI scanning are not detected by humans [2].

Chinese scientists became convinced that infrasound effects were a cause of animal reaction and their earthquake prediction systems include automated recording of nervous budgerigar reactions [5]. This echoes one theory of Turcotte [6] which proposed animals were reacting to acoustic signals inaudible to humans. 

Wadatsumi [7] collected a series of over 1,500 alleged precursors to the Kobe earthquake, but largely refrained from theorizing further. The Kobe (Great Hanshin) earthquake (5.47 a.m., 17 January 1995) had a magnitude M7.3 and caused over 6,000 deaths. Many people in the earthquake zone said in reports to researchers they had awoken about 5 a.m. that day—a definitely unusual time—and about 45 minutes before the earthquake [2,7], so early waking was a possible research subject. Responding to this large earthquake, Ikeya [2] and other papers summarized therein, reported the results of a long series of direct experiments on animals, testing their response to ULF shown by direct experimentation to be generated before rock rupture and hence a possible candidate. In most animals this caused itchiness and/or anxious behaviour [2] and videos on www.eqsigns.net, particularly for the Japanese catfish which detects such disturbances even from the minute fields created by movement of the muscles of prey. The research programme investigated at least 35 species but not humans directly. Mice showed disturbances of circadian rhythm including sleep patterns [8] (confirmed by later Chinese research [9]). The hibernation pattern of snakes and stag beetles was disrupted [2]. One experiment showed direct effects of ULF on isolated frog heart by some mechanism [2]. 

It appeared from the research that smaller animals were more likely to display precursory behavior, so it was thought children might be better subjects than adults and worth investigating. It was theorized that precursory ULF waves might make children anxious and unusually wakeful. This led to some published mention [2] and the present more extensive re-interpretation. 

Although some effect of ULF on animals was strongly supported by these experiments, the other possible rival theories were investigated much less and it cannot be excluded that they contributed to some extent. The catfish sensitivity implied that aerosols were not involved, because they could not affect aquatic species. It remained possible that non-aquatic species might be responding to aerosols or ionized air. 

Unusual smells have long been reported before some earthquakes [10], and are sometimes reported as “like sulphur” (e.g., Christchurch earthquakes, New Zealand, 2010), presumably meaning hydrogen sulphide, but other less definable odours were reported as well. Since human olfactory senses are much less sensitive than those of animals, it is natural to assume animals can sense a variety of trace gases humans cannot. This is presumably not what may be detected by young children. 

It has long been thought that there could be temperature changes in a fault zone before rupture, and another theory might be that animals detect this infrared radiation whereas humans might not [11].

Freund [12] in a series of papers revived the idea of Tributsch that ionized aerosols or similar mechanisms, might be responsible. He and others showed convincingly that stressed rock emitted such ions into the surrounding air through rupture of peroxy bonds in the rock. However his primary emphasis was geophysical. 

Rikitake [13] put forward specific predictions both for time and distance of precursors (including animal behaviour) and earthquake occurrence. These were verified for two large earthquakes in Turkey and Japan [14] *i.e.*, the relationship was not chance. The relationship was further confirmed for New Zealand earthquakes [15], but although there was a clear relationship with animal behavior the mechanism was not further explored. 

During the course of the above papers, other research showed by satellite measurements or derivatives that there are electromagnetic anomalies before earthquakes, and some of these are in the ionosphere itself. Several mechanisms have been proposed for how ground conditions affect the ionosphere but none is yet universally agreed. 

The first author frequently gave talks and lectures in many places in Japan, to classes of children and university students about the geophysics of the Kobe earthquake, reported precursors, his experimental work on ULF and animals, and took the opportunity to gather statistics on early wakening. The data were presented later in a preliminary way [2] but statistical testing was quite limited and there was no other publication. We take this opportunity to explore the implications of the data in greater depth, using different principles. The paper mainly reinforces the idea of statistical correlation, with only a modest examination of the possible mechanisms.

## 2. Methods

The data were collected in two waves. The first was of children 8–10 years old. They were asked whether they awoke before the Kobe earthquake and if so, how much earlier. The second wave, asked similar questions of university students, nearly a decade later, and sought their memories at 8–10 years, *i.e.*, whether they awoke before the earthquake. Although this was in the past, the event was so dramatic and memorable that it seems unlikely much invention occurred. In all cases the location of the respondents was recorded to calculate distance from the epicenter. The interview comprised only the two questions about waking and the location question. There was no attempt to establish norms of waking at 5:47 a.m.; the memories of children for that time would be less reliable than for the earthquake. The analysis relied on internal features of the data accessible though statistical analysis. 

The data were divided into time and distance categories. The two time classes used here were less than 1 minute, and more than 1 minute which was assumed to be a difference that children could detect and report. The other reason for the 1 minute time division is the possibility that respondents were detecting the p-waves ahead of the s-waves, and the former may be taken as travelling at perhaps 6 km sec^−1^. The p- and s-waves for short distances would be within 1 minute of each other. Anything more than one minute might represent a precursor effect. For the three most distant classes, 400–500 km, the time between p- and s-waves could be more than 1 minute but this probably matters little, because only 2 of those children reported early wakening. 

For the closest distance—about 15 km—several of the child respondents had lost relatives, and if this was so there was no insistence on returning the questionnaire, so as to minimize painful memories.

## 3. Results

In a survey like this, the response rate is important, because refusals to answer may significantly impact the results. In many such surveys it has not been possible to control for the response rate, but in the present case because it was a class-room situation, there were 100% returns except for the closest distance, Kobe itself, in which refusals were 5%. A significant number of respondents did not supply wake-up times at all, and although a chi-squared test could not differentiate these from the *t* < 1 data, and they probably were in that class, they were not used. A small sample of data from Kyoto/Okayama was discarded because the small class size was very different from the others, and the error on the respondent percentage was very large.

The results are shown in Table 1.

**Table 1:** The wake-up ratio and percentage of pupils who lived at various distances (km) from the epicenter and the time of waking, *t*, (in minutes) before the Kobe Earthquake.

City	Distance	Ratio ( *t* > 1) (%)	Ratio ( *t* < 1) (%)
Kobe	15	8/110 (7.2%)	16/110 (14.5%)
Nishinomiya	30	16/98 (16.3%)	5/98 (5.1%)
Suita-Takatsuki	50	17/83 (20.5%)	6/83 (7.2%)
Kitano, Osaka	42	27/182 (14.8%)	3/182 (1.6%)
Fukuzaki (W)	46	11/185 (5.9%)	14/185 (7.6%)
Nara (E)	75	25/187 (13.4%)	10/187 (5.3%)
Nagoya	200	2/116 (1.7%)	0/116 (0%)
Hiroshima/Yamaguchi	400	0/55 (0%)	2/55 (3.6%)
Shikoku/Kyushu	400	0/32 (0%)	0/32 (0%)
Tokyo	500	0/31 (0%)	0/31 (0%)

Nearly 1,100 responses were collected.

## 4. Discussion

### 4.1. Exponential Decrease in Results with Distance

There were 62 reports of waking less than a minute before the earthquake, hence significantly fewer than the 106 reports of waking more than a minute before (6 of these were in Kyoto and excluded from Table 1). We also note that the total, 168, is only about 15% of total respondents, so the sensitivity to be explained is a minority phenomenon. The number of children responding to the supposed p-waves at *t* < 1 is actually less than the number responding positively to *t* > 1, hence the *t* > 1 class is unusually interesting. Chi-squared tests showed that the numbers involved for the closer distances were not merely random fluctuations of numbers in those classes. These tests were with the actual numbers rather than fitted functions. A random fluctuation assumption was tested against the assumption that the number reported in each distance class was a similar proportion of the total.

**Figure 1:**
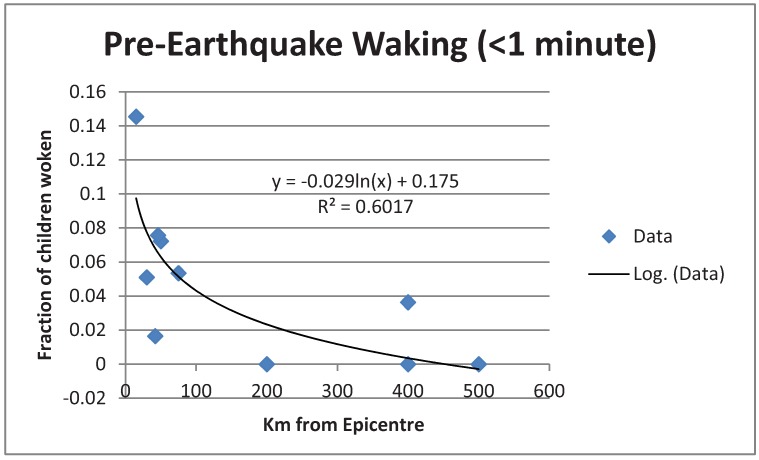
Log fit to data of children who woke less than a minute before the quake.

**Figure 2:**
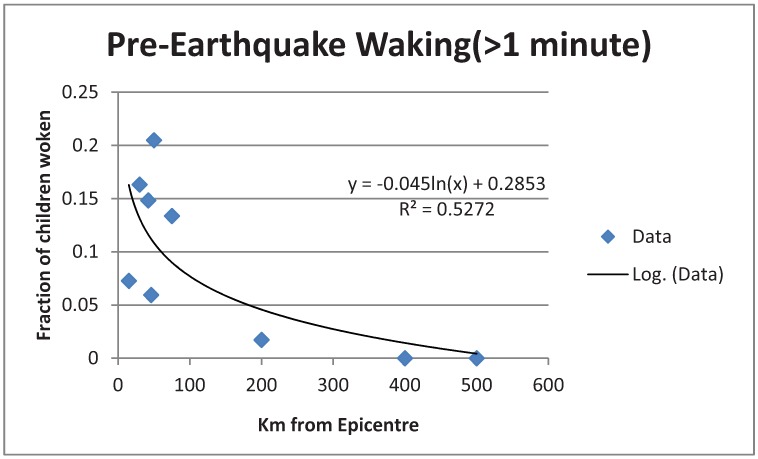
Log fit to data of children who woke more than a minute before the quake.

In Figure 1, Figure 2 are plotted log fits to the two time classes of data. An exponential decrease with distance is shown. Attempts to fit a linear function gave worse fits. 

A chi-squared test showed that the two time classes had data distributions which were significantly different (*p* = 4.7 × 10^−8^) from each other. This suggested different phenomena were being detected.

### 4.2. Psychological Explanation Unlikely

The variances explained were 60% and 53% for Figure 1, Figure 2 respectively, which are unusually high. These are far higher than usually found in a psychological/sociological survey, which normally would only explain about 30–40% of the variance, a sociological rule of thumb. For example as a typical statement [16] “…we should expect to be satisfied with 30% or 40% of the variance accounted for” (p. 103). Variance percentages such as 60% or 53% for purely psychological origins would usually be treated as too high, and not resulting from psychological causes. This is found in the treatment by the above authors [16]. The conclusion is that neither the data from *t* > 1 or *t* < 1, are likely to be psychological inventions solely, and unreliability of memory, which would increase variance, cannot be too severe a factor. Some other factor must be involved as well, such as a physical cause. In the case of data from less than a minute, that might well be reactions to p-waves, but for the other data that is impossible. 

This argument appears to apply to data other than for children. In [15] total replies about all claimed precursors (animals, human, geophysical, meteorological, electronic) to the M7.1 Christchurch (NZ) earthquake (2010), were examined and also found to decrease per head of population with distance in an exponential fashion. These precursors were hours to days before the earthquake, hence not reactions to p-waves. In that paper it was cautioned that the reason for this exponential relationship might be solely psychological. However an exponential fit to the data explained 68% of the variance, which in retrospect can be seen to be far too high for a merely psychological explanation. In that case also, a physical cause must be sought.

### 4.3. Decrease with Distance is not due to a Point/Line Source

Both these classes decrease exponentially with distance, such that the decrease to half the 15 km value as determined from the equations of fit, occurs at 79 and 91 km respectively. For both these values the decrease is unusually slow with distance. The surface length and epicenter depth of fault-line rupture for the Kobe quake were 9 and 16 km respectively [17]. Since the decrease of earthquake strength from an approximately point source 16 km deep is supposed to follow the inverse square law, calculation shows that on the surface it should decrease to half from 15 to only 42 km, much faster than the observed 15 to about 85 km (the corresponding figure for general precursors in the Christchurch study was a large 150 km). This implies that something is affecting the children which is generated not at a point or line source which would correspond to the epicenter of an earthquake, but a very extended source of at least 100 km across. 

An extended source producing a physical effect, is not likely to be a rupture point or fault line, but could be an extended area of stressed rock. Thus one might imagine geoelectromagnetic effects or trace gas release, such as of ^222^Rn. The distances do correspond better with the several tens of kilometers usually detected in displacement/distortion maps pre-/post-earthquake by radar interferometry e.g., [18], however more detailed modeling and better data would be needed for greater clarity and more precise explanation.

Since the percentage of variance explained in plots of percentage *versus* distance is quite high, this means that the exponential relationship is quite good. If there are two components present, such as psychological and physical, they are both producing a good relationship and by coincidence their exponential properties are quite similar. This means for *t* > 1 data, either psychological or physical causes have a half value compared with 15 km data, which is similar, and about 80–90 km. Alternatively it is possible the physical cause greatly predominates, and the effect of psychology is minor. 

Comparing the results from near Kobe to sites a few hundred kilometers away, it seems as though the strength of the effect nearby could be approximately 20× that at many hundred kilometers, and may therefore be classified as a strong effect. 

It should be noted that this treatment assumes a linear reaction of the children to the stimulus, in other words, the percentage of children woken is proportional to earthquake intensity, and this assumption might not be correct. It is a simple first hypothesis, but with large samples starts to become over-simplistic, because reaction of the children at a given distance is likely to be normally distributed, which would produce a sigmoid rather than a linear response at that point. However for smaller samples such as those here, even the sigmoid response is approximately linear. The use of more complex models would be premature, because much larger samples are needed.

It is rather hard to imagine this sensitivity shown by children to be very useful for practical earthquake prediction. Only a minority of children displayed it. In theory an automated CCTV measure of child restlessness might be developed to parallel the automated measurement of budgerigar movement developed by the Chinese for earthquake prediction [5]. This would take considerable effort and resources. It would need a monitoring system in the homes of a significant percentage of the population, and the implied degree of surveillance seems quite unlikely to eventuate.

### 4.4. Parallel Data

Similarly within the data collected for one later report [15] at an early childhood centre in New Zealand by supervisors a day or two before a swarm of earthquakes (2011), there was a claimed clear “increase in parents reporting children not sleeping, waking often in the night and being clingy. This has happened prior to all the big earthquakes, currently parents have started recently to report this again”. “Clingy” means anxious. This type of report was not solicited so may indeed correspond to real sensitivities particularly because they were not aware of the earlier data reported in [2,7].

A parallel report from the same New Zealand data set, again unsolicited, claimed large behaviour disturbances among adult mental patients in long-term hospital care in Christchurch, and another claimed a different historical instance of this in another New Zealand city. These reports are qualitative only, and statistical evaluation is not possible, but could imply some response from adult humans. 

Although there is considerable literature surrounding the qualitative psychological effects of disasters, there are rather few literature reports of the quantitative change of psychological reactions to disasters with distance. This change would be expected on the grounds that people will estimate their personal risk of being affected is less with distance, for example risk from a toxic spill. However this distance estimation does not appear to have been studied in the literature for oil spills, or even an accident as large as the Bhopal methyl isocyanate event. It was surveyed for a Toulouse AZF release [19], the mental effects of which declined with a half value of only about 1.7 km. This is small, but the value will depend on the perception of the type of event, and its perceived dangers. A large event might well have psychological repercussions to a few hundred kilometers. Detailed explanations are not always easy, as shown by the case of the aftermath of the Hiroshima nuclear explosion [20] in which a real effect was seen, but prevalence of two separate psychological effects actually increased within the first few kilometers away from the hypocenter (rather than decreasing) before declining rapidly to background levels. These examples showed such a rapid decline with distance that they tend to support little psychological contribution to the excess waking reported by the children in the Kobe earthquake, *i.e.*, it is more likely a real physiological response. 

However there is known widespread psychological response to supposed radiation exposure in villages surrounding the former Soviet Union nuclear test site in Kazakhstan near Semipalatinsk [21], and the psychological effects are seen over hundreds of kilometers, which corresponds to the local belief structure about fallout in that region. It remains possible therefore that there can be psychological effects at considerable distance. 

### 4.5. The Case of ^222^Rn

One cause of human sensitivity which is unlikely is exposure to ^222^Rn, although its changing concentration near earthquake epicentres is often presented in the literature. This is because the amounts found during common meteorological temperature inversions above cities already exceed by a factor of ten the maximum likely levels found in monitoring areas before earthquakes [22,23]. Any effects of these natural levels of radon, none of them novel, should not be anxiety-producing. 

### 4.6. ULF as a Cause

One possible candidate for some precursory effects on animals or humans remains ULF/ELF (Extremely Low Frequency) radiation. It is known to be created before and during the stressing and fracture of rock in earthquakes [24,25], and the extensive experimental program of Ikeya [2] showed many animals become itchy and anxious when exposed to it. Children were not tested, and there appears to be no prior literature involving them. ULF presence before earthquakes has also been established by satellite observation and by ground observation [2,26]. However there is also much ULF in urban environments and further investigation is needed to establish whether precursors of an earthquake would be strong enough to be novel. Whatever cause is suggested, it should also be able to create effects in the ionosphere over a wide area, and ULF/ELF is more appropriate than many other agents proposed. 

Future research will need to concentrate on exploring more fully the actual physiological effects of the proposed mechanisms—air ions and ULF, *etc*. or search for other explanations of the fairly convincing animal-associated behaviours which have been found. 

Limitations on the data include the possible unreliability of children’s memories, and their estimation of time. Another limitation is that no systematic data from adult humans were collected. However near 100% response to a survey as found in the present case is rare and helpful. 

It was rather novel to derive information about a physical source from a sociological survey. 

## 5. Conclusion

Statistical examination of the children’s wakefulness database shows that a uniquely psychological explanation is unlikely and that a physical cause is more likely, but one that originates in an area very much larger than the several tens of kilometers around the usual earthquake epicenter; rather for an M7.3 earthquake, it is likely to be more than 100 km in diameter. Increased Radon concentration is not likely to be sufficiently novel to be the cause, because it is already frequently and unknowingly experienced at high concentrations particularly in an urban environment under temperature inversion conditions. 

However, any practical use of this information might be restricted to institutions such as orphanages where there could be other reasons to monitor the restlessness of children by automated means. Ionospheric monitoring [26] could be a more objective test, and is probably the way of the future. 

## References

[1] Tributsch H. (1982). When the Snakes Awake.

[2] Ikeya M. (2004). Earthquakes and Animals.

[3] Fleischer R.L., Mogro-Campero A. (1992). ^222^Rn premonitory signals for earthquakes?. EOS Trans. Am. Geophys. Union.

[4] Kirschvink J.L. (2000). Earthquake prediction by animals: Evolution and sensory perception. Bull. Seismol. Soc. Am..

[5] Li J.Z., Bai Z.Q., Chen W.S., Xia Y.Q., Liu Y.R., Ren Z.Q. (2003). Strong earthquakes can be predicted: A multidisciplinary method for strong earthquake prediction. Nat. Haz. Earth Sys. Sci..

[6] Turcotte D.L. (1991). Earthquake prediction. Ann. Rev. Earth Planet. Sci..

[7] Wadatsumi K. (1995). 1519 Statements on Precursors.

[8] Yokoi S., Ikeya M., Yagi S., Nagai K. (2003). Mouse circadian rhythm before the Kobe earthquake in 1995. Bioelectromagnetics.

[9] Li Y., Liu Y., Jiang Z., Guan J., Yi G., Cheng S., Yang B., Fu T., Wang Z. (2009). Behavioral change related to Wenchuan devastating earthquake in mice. Bioelectromagnetics.

[10] Stothers R.B. (2004). Ancient and modern earthquake lights in Northwestern Turkey. Seismol. Res. Lett..

[11] Saraf A.K., Rawat V., Banerjee P., Choudhury S., Panda S.K., Dasgupta S., Das J.D. (2008). Satellite detection of earthquake thermal infrared precursors in Iran. Nat. Haz..

[12] Freund F., Sornette D. (2007). Electro-magnetic earthquake bursts and critical rupture of peroxy bond networks in rocks. Tectonophysics.

[13] Rikitake T. (2001). Prediction and Precursors of Major Earthquakes.

[14] Whitehead N.E., Ulusoy U., Asahara H., Ikeya M. (2004). Are any publicly reported earthquake precursors valid?. Nat. Haz. Earth Syst. Sci..

[15] Whitehead N.E., Ulusoy Ü.  (2013). Macroscopic anomalies before the September 2010 *M* = 7.1 earthquake in Christchurch, New Zealand. Nat. Haz. Earth Syst. Sci..

[16] Bell A.P., Weinberg M.S., Hammersmith S.K. (1981). Sexual Preference: Its Development in Men and Women.

[17] Johansen A., Sornette D., Wakita H., Tsunogai U., Newman W.I., Saleur H. (1996). Discrete scaling in earthquake precursory phenomena: Evidence in the Kobe earthquake, Japan. J. Phys. I France.

[18] Massonnet D., Rossi M., Carmona C., Adragna F., Peltzer G., Feigl K., Rabaute T. (1993). The displacement field of the Landers earthquake mapped by radar interferometry. Nature.

[19] Cohidon C., Diène E., Carton M., Fatras J., Goldberg M., Imbernon E. (2009). Mental health of workers in Toulouse 2 years after the industrial AZF disaster: First results of a longitudinal follow-up of 3,000 people. Soc. Psychiat. Psychiat. Epidemiol..

[20] Yamada M., Izumi S. (2002). Psychiatric sequelae in atomic bomb survivors in Hiroshima. Soc. Psychiat. Psychiat. Epidemiol..

[21] Kawano N., Hirabayashi K., Matsuo M., Taooka Y., Hiraoka T., Apsalikov K.N., Moldagaliev T., Hoshi M.  (2006). Human suffering effects on nuclear tests at Semipalatinsk, Kazakhstan: Established on the basis of questionnaire surveys. J. Radiat. Res..

[22] Liperovsky V.A., Meister C.-V., Liperovskaya E.V., Davidov V.F., Bogdanov V.V. (2005). On the possible influence of radon and aerosol injection on the atmosphere and ionosphere before earthquakes. Nat. Haz. Earth Syst. Sci..

[23] Magalhães M.H., Amaral E.C.S., Sachett I., Rochedo E.R.R. (2003). Radon-222 in Brazil: An outline of indoor and outdoor measurements. J. Envir. Radioact..

[24] Bortnik J., Bleier T.E., Dunson C., Freund F. (2010). Estimating the seismotelluric current required for observable electromagnetic ground signals. Ann. Geophys..

[25] Dunson J.C., Bleier T.E., Roth S., Heraud J., Alvarez C.H., Lira A. (2011). The pulse azimuth effect as seen in induction coil magnetometers located in California and Peru 2007–2010, and its possible association with earthquakes. Nat. Haz. Earth Syst. Sci..

[26] Akhoondzadeh M., Parrot M., Saradjian M.R.  (2010). Electron and ion density variations before strong earthquakes (M > 6.0) using DEMETER and GPS data. Nat. Haz. Earth Syst. Sci..

